# Does colectomy in UC patients impact on the PSC onset?: A national registry brings new insights

**DOI:** 10.1002/ueg2.12392

**Published:** 2023-04-28

**Authors:** Claire Liefferinckx

**Affiliations:** ^1^ Department of Gastroenterology Université Libre de Bruxelles Brussels Belgium

**Keywords:** epidemiology, hepatology, inflammatory bowel disease, primary sclerosing cholangitis, ulcerative colitis

Primary sclerosing cholangitis (PSC) is a chronic liver disease quite rare in the general population but closely related to inflammatory bowel disease (IBD) as 70%–80% of PSC patients have a concomitant IBD.^1^, ^2^ This close relationship between IBD and PSC supports the existence of a gut‐liver axis, where both dysbiosis and a leaky gut may predispose to translocation of microbial components and pro‐inflammatory cytokines to the portal circulation, with secondary biliary epithelial cells and peribiliary inflammation.^3^, ^4^ Hence, the presence of the colon appears a key player in PSC pathogenesis. Several studies reported the deleterious role of colon in situ in the risk of recurrent PSC after transplantation^5^ while colectomy prior to PSC diagnosis was associated with a decreased risk of liver transplantation or death in UC patients.^6^

In the current issue of the United European Gastroenterology Journal, Lundberg Båve et al. addressed the impact of colectomy on the future development of PSC in UC patients.^7^ Based on national registry, the authors performed a matched register‐based cohort study comparing UC patients with and without colectomy to assess the risk of PSC onset. They matched 5.577 UC colectomized patients with 15.078 UC patients with colon in situ for a median follow‐up of 12 years. During the follow‐up, 190 (3.4%) colectomized UC patients and 450 (3.0%) UC comparators developed PSC, without a significant difference between groups. Due to the frequent delay in PSC diagnosis, the authors conducted a sensitivity analysis by moving back 5 years the date of PSC diagnosis but no difference in the incidence of PSC onset between colectomized and UC comparators was observed as well. In sub‐analyses, the risk of PSC onset appeared slightly different between colectomized and comparators patients according to age at UC diagnosis, age at colectomy, year of colectomy and time between UC onset and colectomy. These results lead to the discussion as methodologic biases could influence these divergent observations. First, the absolute numbers of PSC diagnosis in sub‐analysis remained low despite national registry. Second, the registry spanned a large period of time (from 1990 to 2018) which can influence these results. On the one hand, the diagnostic method of PSC has changed over time coupled to a greater awareness of this disease in IBD patients.^8^ On the other hand, the therapeutic management of UC dramatically changed.^9^ A last limit of this study is the lack of knowledge of the colectomy indication, which is a valuable information to improve the interpretation of results. Nonetheless, authors clearly mentioned these limits and the study remains of great value with regards to the available literature.

In conclusion, Lundberg Båve et al. provided new insights on the risk of PSC by showing that colectomy does not impact the PSC onset in UC patients although sub analyses suggested that more granularity is needed in future studies. Overall, this epidemiologic observation is a newly valuable information to add in the understanding of this disease for whom the pathogenesis remains poorly understood.

## CONFLICT OF INTEREST STATEMENT

The author has no conflicts of interest related to this article.

## Data Availability

Data sharing is not applicable to this article as no datasets were generated or analysed during the current study.
